# Synergistic role of c-Myc and ERK1/2 in the mitogenic response to TGFβ-1 in cultured rat nucleus pulposus cells

**DOI:** 10.1186/ar2567

**Published:** 2008-12-05

**Authors:** Tomoko Nakai, Joji Mochida, Daisuke Sakai

**Affiliations:** 1 Division of Organogenesis, Research Center for Regenerative Medicine, Tokai University School of Medicine, Shimokasuya 143, Isehara, Kanagawa, 259-1193, Japan; 2 Department of Orthopaedic Surgery, Surgical Science, Tokai University School of Medicine, Shimokasuya 143, Isehara, Kanagawa, 259-1193, Japan

## Abstract

**Introduction:**

Although transforming growth factor β1 (TGFβ1) is known to be a potent inhibitor of proliferation in most cell types, it accelerates proliferation in certain mesenchymal cells, such as articular chondrocytes and nucleus pulposus cells. The low ability for self-renewal of nucleus pulposus cells is one obstacle in developing new therapeutic options for intervertebral disc diseases, and utilizing cytokines is one of the strategies to regulate nucleus pulposus cell proliferation. However, the precise cell cycle progression and molecular mechanisms by which TGFβ1 stimulates cell growth remain unclear. The aim of this study was to elucidate a mechanism that enables cell proliferation with TGFβ1 stimulation.

**Methods:**

We tested cultured rat nucleus pulposus cells for proliferation and cell cycle distribution under exogenous TGFβ1 stimulation with and without putative pharmaceutical inhibitors. To understand the molecular mechanism, we evaluated the expression levels of key regulatory G_1 _phase proteins, c-Myc and the cyclin-dependent kinase inhibitors.

**Results:**

We found that TGFβ1 promoted proliferation and cell cycle progression while reducing expression of the cyclin-dependent kinase inhibitors p21 and p27, which are downregulators of the cell cycle. Robust c-Myc expression for 2 h and immediate phosphorylation of extra cellular signal regulated kinase (ERK1/2) were detected in cultures when TGFβ1 was added. However, pretreatment with 10058-F4 (an inhibitor of c-Myc transcriptional activity) or PD98059 (an inhibitor of ERK1/2) suppressed c-Myc expression and ERK1/2 phosphorylation, and inhibited cell cycle promotion by TGFβ1.

**Conclusions:**

Our experimental results indicate that TGFβ1 promotes cell proliferation and cell cycle progression in rat nucleus pulposus cells and that c-Myc and phosphorylated ERK1/2 play important roles in this mechanism. While the difference between rat and human disc tissues requires future studies using different species, investigation of distinct response in the rat model provides fundamental information to elucidate a specific regulatory pathway of TGFβ1.

## Introduction

Transforming growth factor β1 (TGFβ1) is known to be a potent inhibitor of proliferation in most cell types, including keratinocytes [1], endothelial cells [2-4] lymphoid cells [5-7] and mesangial cells [8]. Conversely, TGFβ1 stimulates proliferation in certain mesenchymal cells such as bone marrow derived mesenchymal stem cells (BM-MSCs) [9], chondrocytes [10-12] and cells with osteoblastic phenotypes [13]. However, the exact mechanism of stimulation of cell proliferation by TGFβ1 has not been elucidated.

Previous studies suggested that endogenous c-Myc mRNA and protein decrease rapidly when TGFβ1 inhibits cell growth [14-17]. c-Myc is a helix-loop-helix-leucine zipper oncoprotein that plays an important role in cell cycle regulation [18]. It has been also shown that elevated c-Myc activity is able to abrogate the cell cycle suppressing effect of TGFβ1; the mouse keratinocyte cell line (BALB/MK) constitutively expresses endogenous *c-myc*, and showed resistance to the arrest of growth by TGFβ1 [19]. Similarly, *c-myc*-transfected Fisher rat 3T3 fibroblasts showed upregulation in colony formation in soft agar with TGFβ1 treatment [20]. At the same time, these investigators suggested that TGFβ is a bifunctional regulator of cellular growth [19,20].

Considering these findings, we hypothesized that the cells that show mitogenic response to TGFβ1 have a unique mechanism dependent on endogenous c-Myc. We determined the mitogenic effect of TGFβ1 on cultured rat nucleus pulposus cells and whether the small-molecule c-Myc inhibitor, 10058-F4, obstructed cell proliferation caused by exogenous TGFβ1. This inhibitor is a recently identified compound that inhibits the association between c-Myc and Myc-associated factor X (Max). Because c-Myc/Max heterodimers are necessary for binding E-box DNA in the target gene, the interruption of their association inhibits the transcriptional function of c-Myc [21].

Secondly, to suppress expression of c-Myc in protein level, we tested an inhibitor of extracellular signal regulated kinase (ERK)1/2, PD98059 [22]. This was investigated since, it has been reported that mitogen activated protein kinase (MAPK) subtype ERK1/2 mediates TGFβ1 signaling in rat articular chondrocytes [23] and stabilizes c-Myc protein expression [24].

To understand the molecular mechanism of cell cycle regulation by TGFβ1, we utilized western blot analysis. The cell cycle is known to be controlled by positive and negative regulators. The positive regulators are cyclin and cyclin-dependent kinase (CDK) complexes [25]. Cell cycle progression through G_1 _into S phase requires cyclin D-CDK4/6 and cyclin E-CDK2, which phosphorylate the retinoblastoma protein [26]. CDK inhibitors (CKIs) are the negative regulators and are grouped into two families [27]. The INK4 family (p15, p16, p18, p19 and p20) only bind and inactivate cyclin D-CDK4/6 complex, while the Cip/Kip family (p21, p27, and p57) show broader substrate specificity inactivating both cyclin D-CDK4/6 and cyclin E-CDK2 kinase complexes [28]. We examined the expression of p15^INK4^, p21^WAF1/Cip1 ^and p27^Kip1^, which are known to prevent cell cycle progression under the growth inhibitory effect of TGFβ1 [29-32].

The aim of the present study was therefore to reveal the role of c-Myc in mitogenic response to TGFβ1 in nucleus pulposus cells. The study was designed to (1) analyze the effect of TGFβ1 on cell proliferation and the cell cycle progression in nucleus pulposus cells, (2) determine if c-Myc transcription inhibitor obstructed the effect of TGFβ1, and (3) determine the role of ERK1/2 in stabilizing the expression of c-Myc.

## Materials and methods

### Antibodies and reagents

Recombinant human TGFβ1 was obtained from PeproTech Pharmacological (London, UK). Pharmacological c-Myc inhibitor, 10058-F4, ((Z, E)-5-(4-Ethylbenzylidine)-2-thioxothiazolidin-4-one), which inhibits c-Myc transcriptional activity was supplied by Calbiochem (Darmstadt, Germany). Pharmacological MAPK/ERK kinase inhibitor PD98059 was from Upstate (Lake Placid, NY, USA). Polyclonal rabbit antibodies against rat phospho-MAPK (ERK1/2) (Thr202/Tyr204), p44/42 MAPkinase (ERK1/2), and p27 Kip1 were from Cell Signaling Technology (Beverly, MA, USA). Polyclonal rabbit antibodies against rat p15 INK4b, p21 WAF1/Cip1 and c-Myc were from Abcam (Cambridge, UK) and monoclonal mouse antibody for beta-Actin was from Sigma-Adrich Corp. (St Louis, MO, USA).

### Cell culture

All animal experiments were performed with approval from the Tokai University animal study institutional review board (No.073008). A total of 14 female Sprague-Dawley rats (12 months old; CLEA Japan Inc., Tokyo, Japan) were utilized for the entire study and the cells from at least 3 animals were applied to each experiment. Cryopreserved primary passage rat epidermal keratinocytes were obtained from Cell Applications Inc. (San Diego, CA, USA) and maintained in growth medium (Cell Applications Inc.). Cells from rat intervertebral disc tissues were isolated and processed as previously described [33]. Briefly, the nucleus pulposus was harvested from coccygeal discs of rats and suspended in Dulbecco's phosphate-buffered saline (DPBS; DS Pharma Biomedical, Osaka, Japan) with 0.05% trypsin/0.53 mM Ethylenediaminetetraacetic acid (EDTA; Gibco Invitrogen Corp., Carlsbad, CA, USA) added to achieve final concentrations of 0.01% trypsin and 0.1 mM EDTA and allowed to digest at 37°C for 15 min. Chondrocytes from articular cartilage were prepared following the method of Tukazaki *et al*. [10]. Cartilage slices from knee joints of rats were digested with 0.05% trypsin and 0.53 mM EDTA (Gibco Invitrogen) at 37°C for 30 min, followed by 0.3 mg/mL collagenase P (Roche Diagnostics GmbH, Mannheim, Germany) at 37°C for 4 h. The isolated nucleus pulposus cells and articular chondrocytes were cultured in Dulbecco's modified Eagle medium: Nutrient Mixture F-12, 1:1 Mixture (DMEM/F-12) (Wako Pure Chemical Industries Ltd., Osaka, Japan), containing 10% fetal bovine serum (FBS; Gibco Invitrogen), 100 U/mL penicillin (Gibco Invitrogen) and 100 μg/mL streptomycin (Gibco Invitrogen), at 37°C in 5% CO_2 _humidified atmosphere. The medium was replaced twice a week and the cells were trypsinized and subcultured before the cultured cells reached confluency. The nucleus pulposus has been reported to consist of at least two major cell populations, notochordal cells and chondrocyte-like cells [34,35]. Because cells obtained from the rat disc tissues were variable in morphology until the second passage, we expanded the culture to the third or fourth passage to prepare enough number of the morphologically uniformed cells from each animal. Conversely, because articular chondrocytes were morphologically uniform since primary culture, the second passage was used for the experiments. With regard to keratinocytes, they will not proliferate if keratinization is triggered by passage. Therefore, the primary culture was applied for the experiment in the medium specified by the supplier. Nucleus pulposus and articular chondrocytes were subjected to the experiments using Opti Minimum Essential Medium (Opti-MEM, Gibco Invitrogen). Serum deprivation was performed with 24 h incubation with medium containing 2% FBS followed by 2 h incubation with medium containing 0.5% FBS; 0.5% FBS was fed to maintain cell adhesion throughout every experimental period. All experiments were performed at least three times to confirm consistency.

### Reverse transcriptase-polymerase chain reaction (RT-PCR)

Cells cultured in serum-deprived medium were treated with and without 5 ng/mL TGFβ1 for 24 h. The cells were then harvested and total RNA was isolated using the SV Total RNA Isolation System (Promega, Madison, WI, USA), which included DNase digestion and spin column purification. Primers for rat *c-myc, p15*, *p21*, *p27 *and *β-actin *were designed based on the coding sequences from GenBank ([Genbank:BC091699, AF474979, BC100620, NM_031762, NM_031144] respectively), and synthesized by Invitrogen. For *c-myc *the primers used were CAACGTCTTGGAACGTCAGA (forward) and CTCGCCGTTTCCTCAGTAAG (reverse). For *p15 *the primers used were CAGAGCTGTTGCTCCTCCAC (forward) and CGTGCAGATACCTCGCAATA (reverse). For *p21 *the primers used were AGCAAAGTATGCCGTCGTCT (forward) and ACACGCTCCCAGACGTAGTT (reverse). For *p27 *the primers used were ATAATCGCCACAGGGAGTTG (forward) and CCAGAGTTTTGCCCAGTGTT (reverse). For *β-actin*, the primers were AGCCATGTACGTAGCCATCC (forward) and CTCTCAGCTGTGGTGGTGAA (reverse). For each sample, 2 μg of total RNA was reverse transcribed into cDNA using MultiScribe Reverse Transcriptase (Applied Biosystems, Foster City, CA, USA) and oligo(dT) primers (Applied Biosystems). For PCR 5 μL of cDNA template was amplified in a 25-μL reaction volume of GeneAmp PCR buffer (Applied Biosystems), containing 5.5 mM MgCl_2_, 200 μM of each dNTP, 0.5 μM of appropriate primer pairs and 1 unit of AmpliTaq Gold DNA polymerase (Applied Biosystems). The reaction mixture was kept at 95°C for 10 min for a 'hot-start', followed by PCR of 31 cycles for *p15*, 28 cycles for *p21*, 27 cycles for *p27*, 30 cycles for *c-myc *and 26 cycles for *β-actin*. Each cycle included denaturation at 95°C for 15 s, followed by annealing and extension at 61°C for 1 min. A total of 10 μL of each PCR product was applied to 3% agarose gel for electrophoresis. Resolved bands on the gels were visualized with ethidium bromide on a densitograph system (ATTO Biotechnologies Inc., Tokyo, Japan).

### Cell proliferation assay

To determine cell proliferation, nucleus pulposus cells were plated in 96-well plates at a density of 3,000 cells/well. The cells were allowed to adhere for 24 h in OptiMEM containing 2% FBS. The medium was replaced with OptiMEM containing 0.5% FBS and recombinant human TGFβ1 in final concentrations of 0 (control), 5, or 20 ng/mL. For experiments using pathway specific inhibitors, appropriate concentrations of 10058-F4 or PD98059 were added to the medium as concentrated stock solutions dissolved in dimethyl sulfoxide (DMSO, Wako). The solvent alone was added at 0.08% to serve as the vehicle control. During the 6 days of culture, the culture media were replaced on day 3 with the appropriate medium. After cultivation for the scheduled period, cell numbers were determined using the 3-(4,5-dimethyl-2-thiazolyl)-2,5-diphenyl-2*H*-tetrazolium bromide (MTT; Wako) assay [36]. Briefly, the culture medium was replaced with 0.1 mL of MTT solution (0.5 mg/mL MTT) in serum-free DMEM without phenol red (Gibco Invitrogen). The cells were incubated at 37°C for 2 h, and then the MTT solution was replaced by 0.2 mL of solubilizer solution (80% isopropanol; 20% DMSO; 4% Tween 20) and mixed. The absorbance at 562 nm was determined using a microplate reader (SPECTRA MAX 250, Molecular Devices, Sunnyvale, CA, USA). The cell number was calculated based on the absorbance according to a standard curve of rat nucleus pulposus cells prepared prior to the experiments. The wells for each experimental condition were replicated five times and the representative results from three individual experiments were shown.

### Cell cycle analysis by fluorescence-activated cell sorting (FACS)

The cells were trypsinized, washed and seeded in 25 cm^2 ^flasks at 1 × 10^5 ^cells/flask. The cells were allowed to adhere for 24 h in medium containing 2% FBS. The culture medium of each flask was then replaced with medium containing 0.5% FBS. The appropriate concentrations of 10058-F4 or PD98059 were then added to this medium as concentrated stock solutions dissolved in DMSO. After incubation for 2 h, TGFβ1 (5 or 20 ng/mL) was added to the cultures. After an additional incubation period of 24 h, cell cycle distribution of the nucleus pulposus cells was analyzed by FACS after DNA staining with propidium iodide using the CycleTEST™ PLUS (BD PharMingen, San Diego, CA, USA) kit. CELLQuest (BD PharMingen) and ModiFit LT (BD PharMingen) software was used for calculations of cell acquisition and analysis. Each experiment was duplicated and the results from three individual experiments were shown.

### Western blot

The cells were lysed in ice-cold cell lysis buffer (50 mM Tris/HCl, pH7.5; 2 mM CaCl_2_; 1% TritonX-100) containing protease and phosphatase inhibitors (0.5 mM phenylmethylsulfonyl fluoride (PMSF); 1/50 Complate, a protease inhibitor cocktail (Roche Molecular Biochemicals, Mannheim, Germany); 1 mM Na_3_VO_4 _and 1 mM NaF). Cell lysates were sonicated for 10 s to shear the DNA, then centrifuged at 10,000 *g *for 10 min at 4°C. The supernatant was collected and its total protein concentration was determined using the DC Protein Assay Reagent (Bio-Rad, Hercules, CA, USA). Equal amounts of protein were diluted with sodium dodecyl sulfate (SDS) sample buffer, (reducing conditions were used only for p21) boiled for 5 min, and electrophoresis performed using SDS-polyacrylamide gel electrophoresis (SDS-PAGE). The protein bands separated in the gel were electrotransferred by electroblotting to a polyvinylidene difluoride (PVDF) membrane filter (Bio-Rad). The membrane was then blocked with 3% w/v bovine serum albumin (BSA, Serologicals, Kankakee, IL, USA) in Tris-buffered saline/Tween (TBST: 50 mM Tris, pH 7.6; 150 mM NaCl; 0.1% Tween-20) for 1 h at room temperature. Incubation with the indicated primary antibodies overnight at 4°C in 1% BSA in TBST followed this step. After washing in TBST, the membrane was incubated with secondary anti-IgG antibody conjugated with horseradish peroxidase (Amersham Life Science, Arlington Heights, IL, USA) for 1 h at room temperature. The signals were detected using enhanced chemiluminescence reagent (ECL Plus, Amersham Pharmacia Biotech, Bjorkgatan, Sweden).

### Statistical analysis

The data are presented as the mean and standard error of the mean (SEM). Statistical analysis was performed basically by non-repeated measures analysis of variance (ANOVA) except for the cell cycle experiment, where repeated measures ANOVA was used. When a p-value of < 0.05 was found, the Student-Newman-Keuls test for multiple pair comparisons was used. **Indicates highly significant differences (p < 0.01), * indicates significant differences (p < 0.05) throughout.

## Results

### Different response to TGFβ1 treatment in c-Myc mRNA expression dependent on cell type

**Figure 1:**
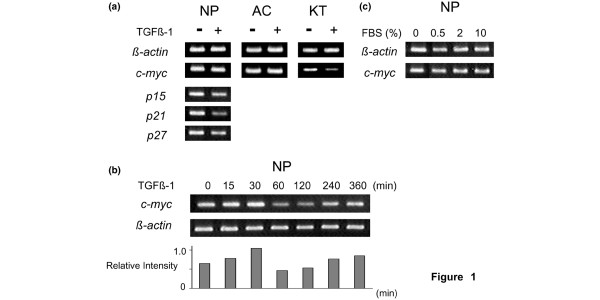
**Effect of transforming growth factor β1 (TGFβ1) treatment on mRNA expression in different cell types (a), Cells were treated with or without 5 ng/mL TGFβ1 for 24 h**. The expression of *c-myc *in nucleus pulposus cells (NP), in articular chondrocytes (AC) and keratinocytes (KT) are presented. The expression of *p15*, *p21 *and *p27 *in NP was also determined. Time course of *c-myc *expression in NP treated with 5 ng/mL TGFβ1 (b). The graph shows the relative intensities of *c-myc *bands normalized for *β-actin *levels by densitographic analysis. Incubation for 24 h with medium containing various concentrations of fetal bovine serum (FBS) did not alter the level of *c-myc *expression in NP (c). The reverse transcription-polymerase chain reaction (RT-PCR) was performed on total RNA extracted from the cells. *β-actin *was used as an internal control.

To investigate endogenous c-Myc mRNA expression and the influence of TGFβ1 treatment on cells derived from different organs, we analyzed gene expression in rat keratinocytes, nucleus pulposus cells, and articular chondrocytes. As shown in Figure 1a, c-Myc mRNA decreased in rat keratinocytes with TGFβ1 treatment, while it was unchanged in nucleus pulposus cells and articular chondrocytes. Further analyses of nucleus pulposus cells indicated that levels of p21 mRNA decreased with TGFβ1 treatment and that levels of c-Myc mRNA were downregulated at the 60 and 120 min time points (Figure 1b). Differences in concentration of FBS in the medium did not alter the expression of c-Myc mRNA in nucleus pulposus cells (Figure 1c).

### TGFβ1 treatment enhanced the proliferation of nucleus pulposus cells

**Figure 2:**
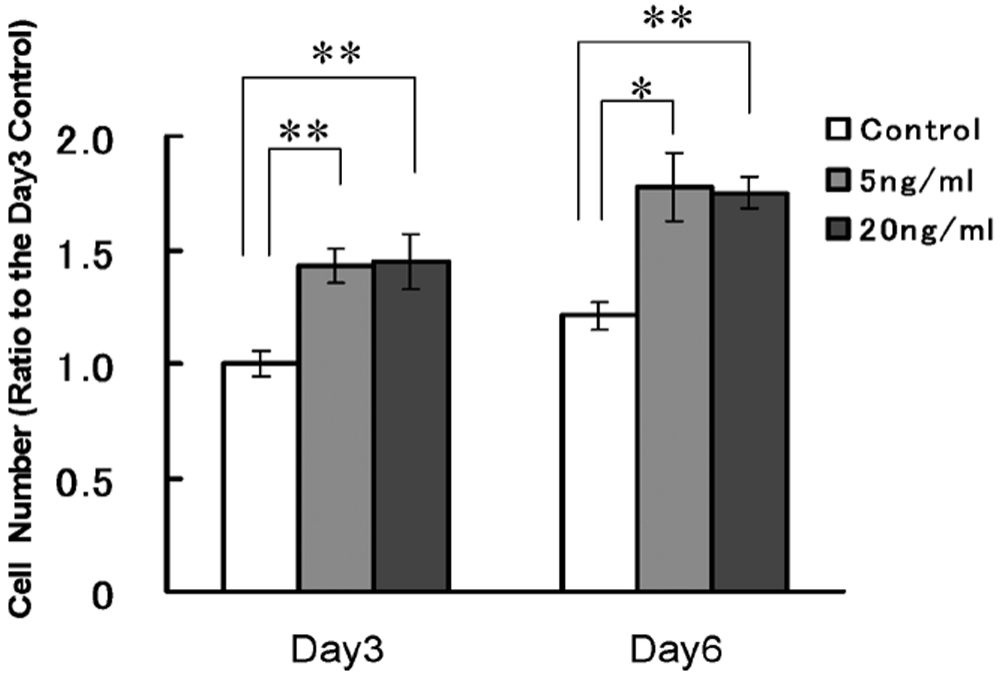
**Nucleus pulposus cell proliferation is upregulated by TGFβ1 treatment**. Cells were plated in 96-well plates in medium containing 2% fetal bovine serum (FBS) for 24 h. This medium was replaced with medium containing 0.5% FBS and cells were treated with 5 or 20 ng/mL transforming growth factor β1 (TGFβ1). Cell proliferation was evaluated by the 3-(4,5-dimethyl-2-thiazolyl)-2,5-diphenyl-2H-tetrazolium bromide (MTT) assay on days 3 and 6 after treatment. Five replicates per experimental condition were made. Data are normalized to values obtained for cells cultured for 3 days in 0.5% FBS containing medium and shown as mean ± standard error of the mean (SEM) (*p < 0.05, **p < 0.01).

To determine the effect of TGFβ1 on cell proliferation, cell number was measured at the given time intervals. Treatment was with either 5 or 20 ng/mL TGFβ1 upregulated cell proliferation on days 3 and 6 (up to 160% compared to the day 3 control (Figure 2)). The statistical significance among the groups in this proliferation assay by ANOVA was p = 4.408E-7. The significances of individual differences by the multiple pair comparisons are shown in Figure 2 (**p < 0.01, *p < 0.05).

### Influence of pathway inhibitors blocked cell growth under TGFβ1 stimulation

**Figure 3:**
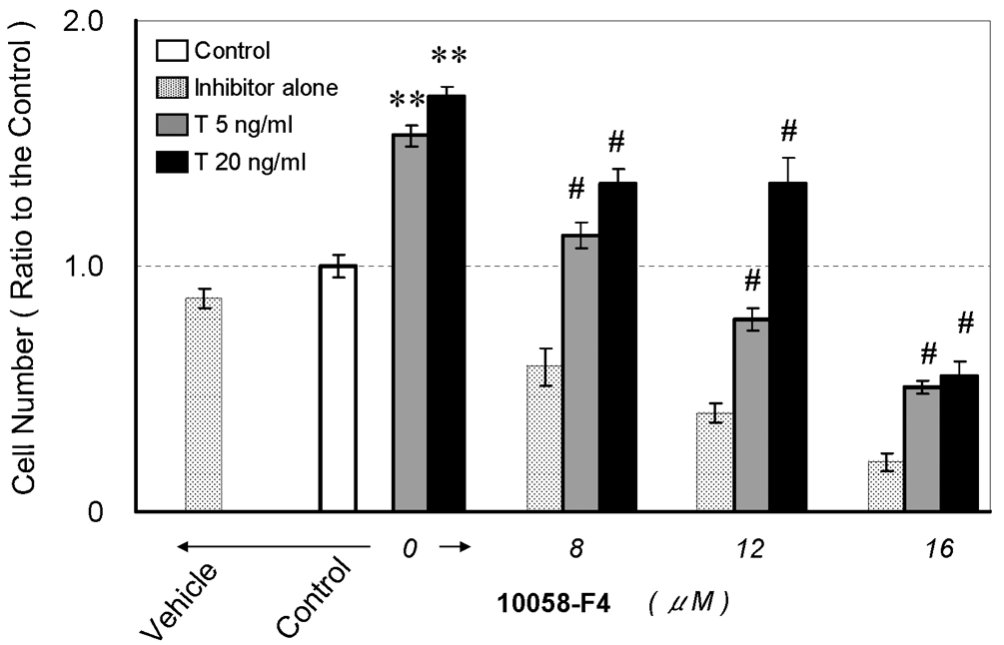
**c-Myc transcription inhibition prevents transforming growth factor β1 (TGFβ1)-stimulated cell proliferation**. Serum-deprived cells in 96-well plates were treated with 5 or 20 ng/mL TGFβ1 (abbreviated to T) with or without 8, 12, 16 μM 10058-F4. Cell proliferation was evaluated by the 3-(4,5-dimethyl-2-thiazolyl)-2,5-diphenyl-2H-tetrazolium bromide (MTT) assay on day 3 after treatment. Five replicates per experimental condition were made. Data are normalized to values obtained for untreated cells cultured in 0.5% serum containing medium and represented as mean ± standard error of the mean (SEM) (**p < 0.01 when compared with control, #p < 0.01 when compared with the TGFβ1-treated group).

As nucleus pulposus cells maintained c-Myc mRNA expression under TGFβ1 stimulation (Figure 1a,b), we hypothesized that c-Myc plays a central role in TGFβ1 signaling for cell growth stimulation. Additionally, to examine the possibility of involvement of the MAPK pathway in regulation of c-Myc stability, we devised serial experiments using the pathway specific inhibitors 10058-F4, an inhibitor of c-Myc transcriptional activity, and PD98059, an inhibitor of extracellular signal regulated kinase (ERK1/2). As shown in Figure 3, 5 or 20 ng/mL TGFβ1 treatment increased the nucleus pulposus cell number (up to 160%, p < 0.01) compared with control. Pretreatment with the c-Myc inhibitor, 10058-F4, caused a dose-dependent significant decrease in cell number (from 32% to 79%, compared with the TGFβ1-treated group, p < 0.01). The 20-ng/mL TGFβ1-treated cultures showed higher resistance to the inhibitory effect of 10058-F4 (8 and 12 μM) than 5 ng/mL TGFβ1. The statistical significance of this experiment using 10058-F4 was p = 1.116E-18.

**Figure 4:**
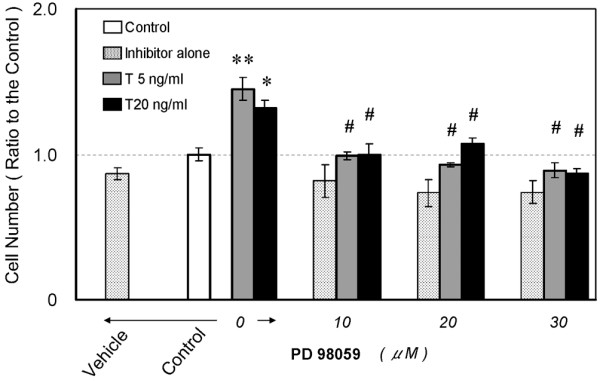
**The inhibition of extracellular signal regulated kinase (ERK)1/2 phosphorylation prevents transforming growth factor β1 (TGFβ1)-stimulated cell proliferation**. Serum-deprived cells in 96-well plates were treated with 5 or 20 ng/mL TGFβ1 (abbreviated to T) with or without 10, 20, 30 μM PD98059. Cell proliferation was evaluated by the 3-(4,5-dimethyl-2-thiazolyl)-2,5-diphenyl-2H-tetrazolium bromide (MTT) assay on day 3 after treatment. Five replicates per experimental condition were made. Data are normalized to values obtained for untreated cells cultured in 0.5% serum containing medium and represented as mean ± standard error of the mean (SEM) (*p < 0.05, **p < 0.01 when compared with control, #p < 0.01 when as compared with TGFβ1-treated group).

Similar results from the cell proliferation assay using the ERK1/2 inhibitor (Figure 4), demonstrated that while treatment with 5 or 20 ng/mL TGFβ1 increased the nucleus pulposus cell number (up to 130% compared with control, p < 0.05), pretreatment with the ERK1/2 inhibitor, PD98059, caused a significant decrease in cell number (from 66% to 76% compared with TGFβ1-treated group, p < 0.01). In contrast to the 10058-F4 results, the differences were not clearly dose-dependent. The statistical significance of this experiment using PD98059 was p = 1.334E-8.

### Effects of TGFβ1 and pathway inhibitors on cell cycle distribution in nucleus pulposus cells

**Figure 5:**
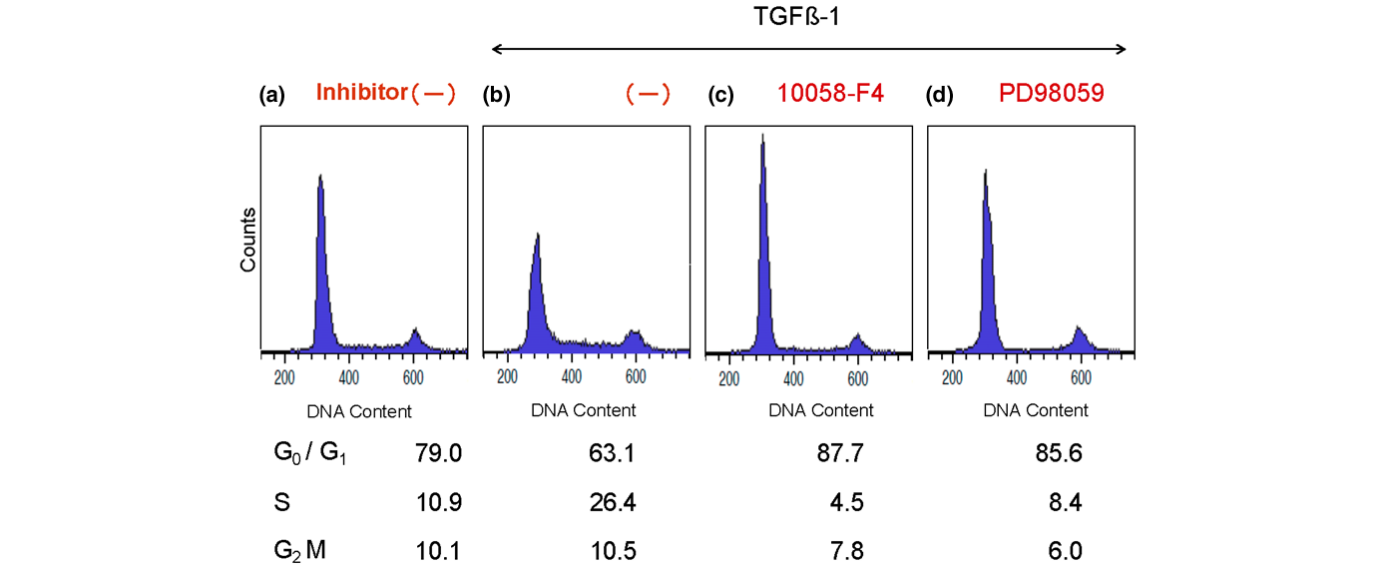
**Cell cycle distribution of nucleus pulposus cells**. Serum-deprived nucleus pulposus cells were cultured with no supplements for 24 h (a). The cells were treated with 5 ng/mL transforming growth factor β1 (TGFβ1) for 24 h (b). At 2 h before the addition of TGFβ1, the cells were treated with 16 μM 10058-F4 (c), or with 30 μM PD98059 (d). The cells were harvested 24 h after the addition of TGFβ1 and the nuclei were stained with propidium iodide. DNA histograms were generated using flow cytometry. Each plot represents the analysis of 10,000 events. The histograms present typical results and the percentage of cells in G_0_/G_1_, S and G_2_/M phases are shown as the average of duplicated measurements.

**Figure 6:**
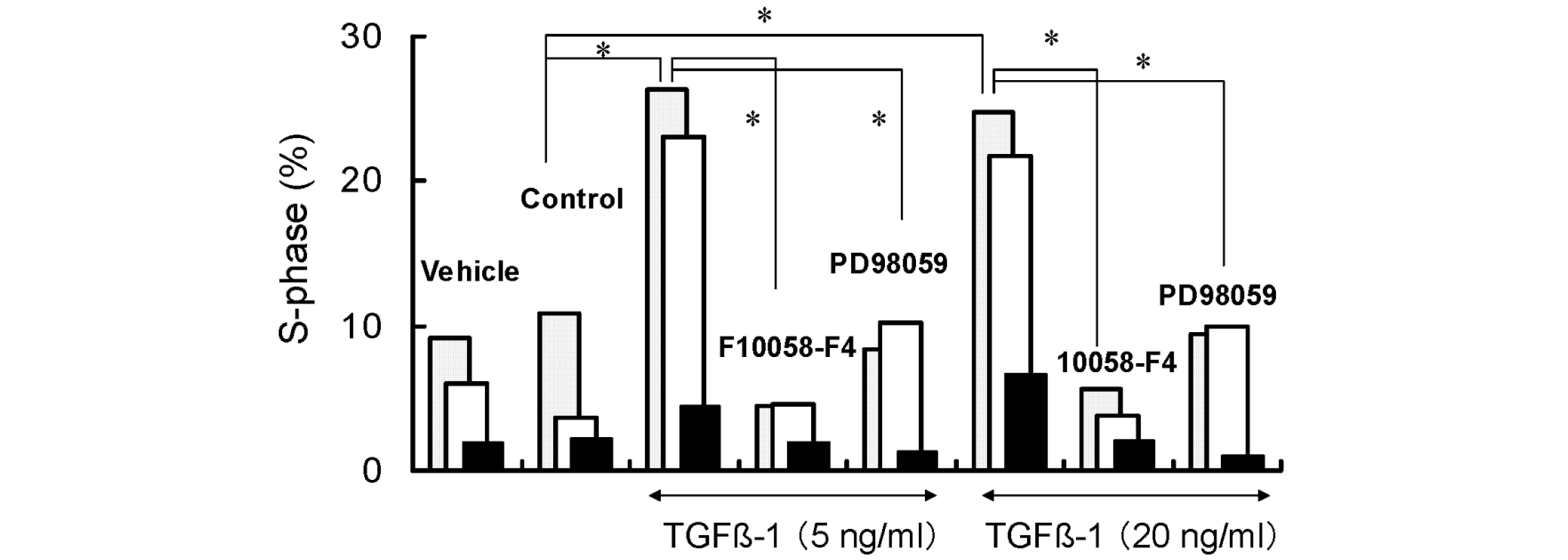
**Effects of inhibitors and transforming growth factor β1 (TGFβ1) on cell cycle progression**. Serum-deprived nucleus pulposus cells were treated with or without inhibitors (16 μM 10058-F4, or 30 μM PD98059) then treated with 5 or 20 ng/mL TGFβ1 for 24 h. The percentage of cells in S-phase was determined with fluorescence-activated cell sorting (FACS). Black bar, white bar and gray bar indicate the results obtained for three rats respectively. (*p < 0.05)

We then used flow cytometry to determine cell cycle progression by quantifying DNA. Effects of inhibition of c-Myc transcriptional activity and inhibition of ERK1/2 activity in the presence of 5 ng/mL TGFβ1 were determined. After serum deprivation, 79.0% of nucleus pulposus cells were in the G_0_/G_1 _phase, 10.9% in the S phase, and 10.1% in the G_2_/M phase (Figure 5a). Treatment with TGFβ1 for 24 h (Figure 5b) significantly increased the percentage of cells in the S phase to 26.4%, indicating that TGFβ1 did not cause cell cycle arrest but acted as a mitogen, unlike its action in some other cell types. In contrast, marked decrease in the percentage of cells in the S phase were observed in the presence of 10058-F4, 4.5% (Figure 5c) or PD98059, 8.4% (Figure 5d). In addition, increase in the G_0_/G_1 _phase were found when cells were treated with these inhibitors (87.7% (Figure 5c) and 85.6% (Figure 5d), respectively), compared to control (79.0% (Figure 5a)). This indicates that these inhibitors have caused cell cycle arrest in the G_0_/G_1 _phase even with treatment with TGFβ1. The results obtained from three different rats are shown in Figure 6. Although the percentages of cells in the S phase differ among individuals, these inhibitors both seem to block the mitogenic effect of TGFβ1 completely. The statistical significance by the repeated measures ANOVA of the cell cycle experiment was p = 3.213E-3.

### TGFβ1 did not abolish c-Myc expression but decreased CDKIs p21 and p27

**Figure 7:**
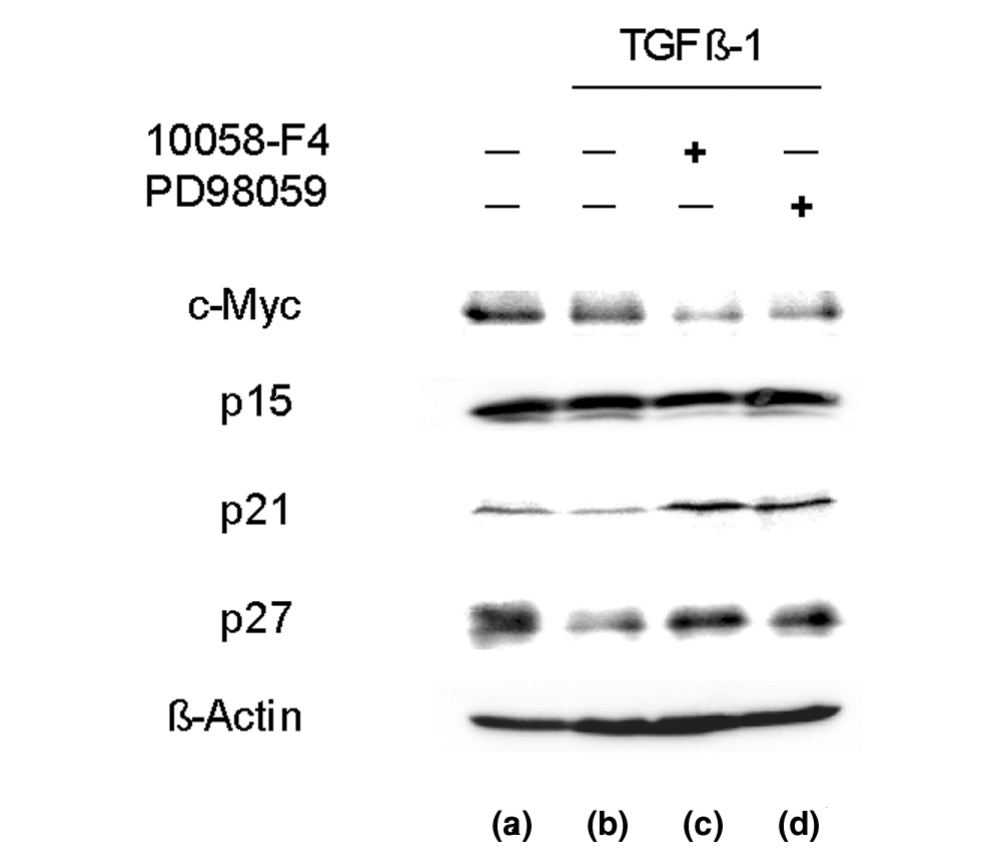
**Western blot analysis of cell cycle regulators**. After 24 h incubation in a medium containing 2% fetal bovine serum (FBS), this medium was replaced with medium containing 0.5% FBS. Nucleus pulposus cells were cultured with no supplements for an additional 24 h (a). The cells were treated with 5 ng/mL transforming growth factor β1 (TGFβ1) for 24 h (b). At 2 h before the addition of TGFβ1, the cells were treated with 16 μM 10058-F4 (c), or with 30 μM PD98059 (d). The cells were harvested 24 h after the TGFβ1 treatment and lysed. Aliquots of the lysates were electrophoresed on 12.5% sodium dodecyl sulfate polyacrylamide gel electrophoresis (SDS-PAGE). The protein bands were blotted to a polyvinylidene diflouride (PVDF) membrane and probed with antibodies against c-Myc, p15, p21, and p27. β-Actin was used as a quantity loading control. Treatment with TGFβ1 without inhibitors (b) did not abolish c-Myc expression but decreased the level of cyclin-dependent kinase inhibitors (CKIs) (p21, p27) compared to the control, while treatments with inhibitors (c, d) diminished c-Myc and upregulated p21 and p27. In contrast, p15 levels were unchanged by any of these treatments.

In parallel experiments, we evaluated the expression levels of key regulatory G1 phase proteins c-Myc, p15, p21 and p27 utilizing western blotting. As seen in Figure 7, TGFβ1 treatment (b) did not abolish c-Myc expression, but pretreatment with either 10058-F4 (c) or PD98059 (d) diminished the level of expression. In contrast, TGFβ1 treatment showed the lowest levels of p21 and p27 when compared with other experimental conditions. Note that pretreatment with either 10058-F4 or PD98059 upregulated the levels of p21 and p27 compared to TGFβ1 treatment. However, no distinguishable change was observed in p15 expression.

### Mitogenic effect of TGFβ1 is supported by coexpression of c-Myc and phospho-ERK1/2

**Figure 8:**
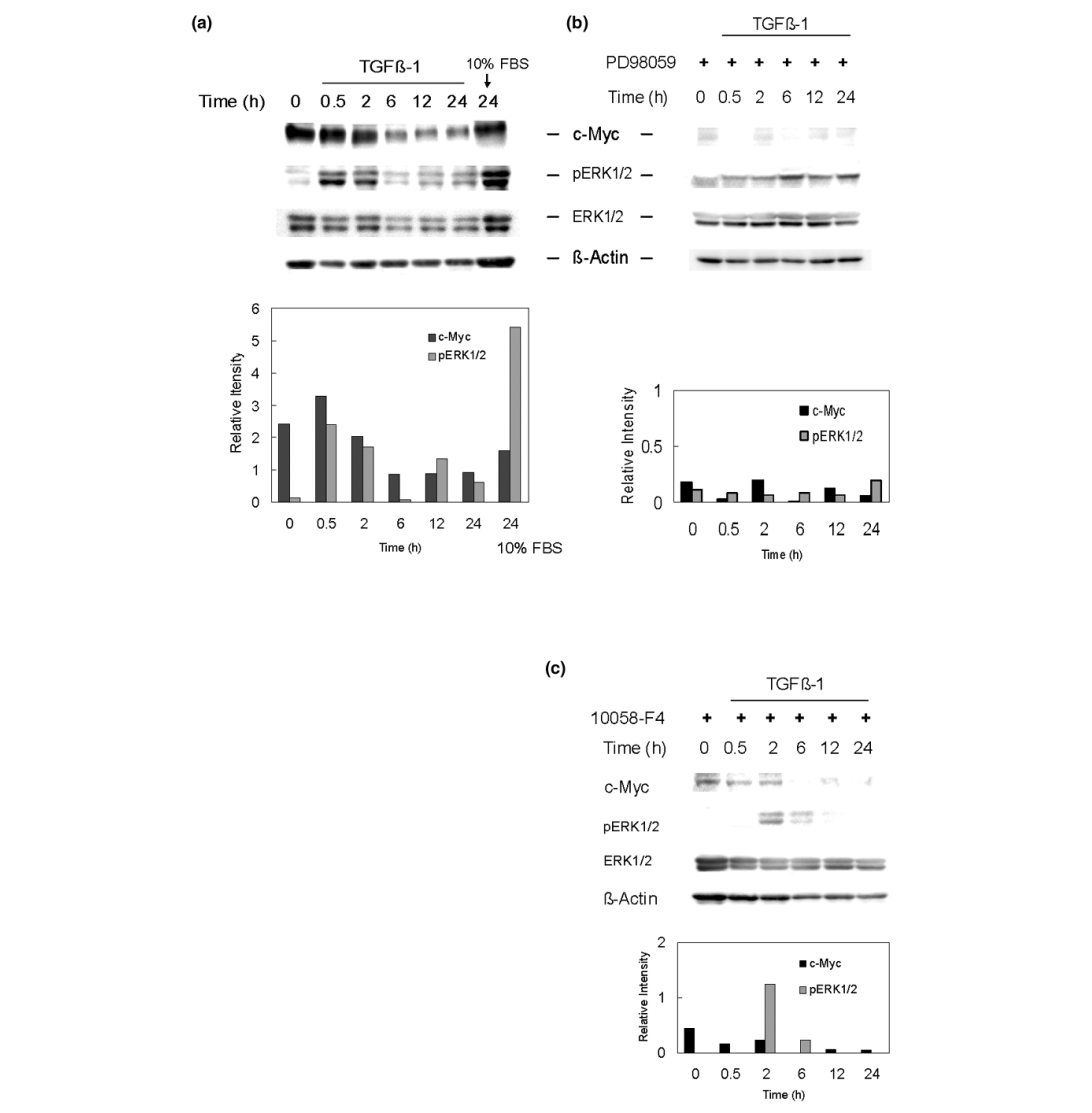
**Time course study of c-Myc and phospho-extracellular signal regulated kinase (ERK)1/2 expression by western blot analysis**. Serum-deprived nucleus pulposus cells were treated with or without 16 μM 10058-F4 or 30 μM PD98059 before the addition of 5 ng/mL transforming growth factor β1 (TGFβ1). The cells were harvested at the times indicated and lysed. Aliquots of the lysates were electrophoresed on 5% to 20% gradient sodium dodecyl sulfate polyacrylamide gel electrophoresis SDS-PAGE). The protein bands were blotted to a polyvinylidene diflouride (PVDF) membrane and probed with antibodies against c-Myc, total ERK1/2 and phospho-ERK1/2. β-Actin was used as a quantity loading control. (a) TGFβ1 treatment induced immediate phosphorylation of ERK1/2 with robust c-Myc expression for 2 h. The expression of c-Myc, phospho-ERK1/2, and total ERK1/2 were detected throughout the experimental period. The right lane indicates the result of 24 h treatment with 10% FBS; c-Myc and phospho-ERK1/2 appear distinctly. (b) Pretreatment with ERK1/2 inhibitor 30 μM PD98059 diminished the expression of c-Myc and interrupted the phosphorylation of ERK1/2. Note that a single isoform corresponding to phospho-ERK2 was detected at all times. (c) Pretreatment with c-Myc inhibitor 16 μM 10058-F4 diminished c-Myc expression and limited ERK1/2 phosphorylation for a short time under TGFβ1 stimulation. Graphs show relative intensities in expression of c-Myc normalized to *β-actin *levels and in expression of phospho-ERK1/2 normalized to total ERK1/2 levels, respectively.

To understand the molecular mechanism underlying TGFβ1-mediated cell cycle modulation, we performed a time-course study on c-Myc and phospho-ERK1/2. Serum-deprived cells were pretreated with or without 10058-F4 or PD98059 then treated with TGFβ1 for different time periods. The cells were harvested and whole cell lysates were analyzed for the expression of c-Myc, phospho-ERK1/2, and total ERK1/2 by western blot. Robust c-Myc expression from the beginning was suppressed at 6 h and ERK1/2 was immediately phosphorylated (activated) by 0.5 to 2 h in TGFβ1-treated preparations (Figure 8a). Both c-Myc and phospho-ERK1/2 were detected throughout the experimental period. The lane on the far right indicates the result of 24 h treatment with 10% FBS in which c-Myc and phospho-ERK1/2 appear distinctly (Figure 8a). These data indicate that coexpression of c-Myc and phospho-ERK1/2 correlates with vigorous cell proliferation.

By contrast, pretreatment with the ERK inhibitor PD98059 diminished the expression of c-Myc and mainly blocked the phosphorylation of ERK1 induced by TGFβ1 treatment (Figure 8b). A single isoform corresponding to phospho-ERK2 was detected at all time points; this suggests that c-Myc expression under TGFβ1 stimulation requires activated ERK1/2, especially ERK1. Similarly, pretreatment with the c-Myc inhibitor 10058-F4 unexpectedly decreased c-Myc expression and interrupted the phosphorylation of ERK1/2 induced by TGFβ1 (Figure 8c). The expression of phospho-ERK1/2 was delayed until the 2-h time point and disappeared after 12 h in spite of coexistent TGFβ1. These data indicate that the inhibition of c-Myc transcriptional activity diminished the level of c-Myc protein itself and also downregulated the phosphorylation (activation) of ERK1/2.

The results of these blot analyses reveal that the effect of the TGFβ1 signal can be mitogenic when c-Myc and phospho-ERK1/2 are both expressed in nucleus pulposus cells.

## Discussion

Although TGFβ1 is a potent inhibitor of growth in most cell types, it has been shown to stimulate growth of certain mesenchymal cells in culture, such as mouse BM-MSCs [9], rat and avian articular chondrocytes [10,11,23,37], human nasal septal chondrocytes [12], and cells with an osteoblastic phenotype from rat parietal bone [38] and from calvariae of 1-day-old mice [13]. In these previous investigations, growth stimulation was shown by upregulation in proliferation or in [^3^H]-thymidine uptake. With regard to intervertebral disc cells, the enhancement of colony formation of human annulus fibrosus cells and increase in density of nucleus pulposus cells in three-dimensional scaffold cultures have been reported [39,40].

In the present study, we found that TGFβ1 significantly stimulated growth of nucleus pulposus cells (Figure 2). To ascertain the effects of TGFβ1, we examined the cell cycle regulatory effect of TGFβ1 in rat nucleus pulposus cells *in vitro*.

TGFβ1 regulates gene expression through Smad transcription factors [41-43]. When TGFβ1 inhibits cell growth, a rapid decrease in endogenous c-Myc mRNA and protein has been observed [14-17]. c-Myc is a transcription factor that promotes cell growth and proliferation, and under certain conditions, apoptosis, and tumor cell immortalization [44]. Levels of c-Myc are increased or decreased in response to mitogenic or growth inhibitory stimuli, respectively [17]. It is notable that *c-myc *transfected Fisher rat 3T3 fibroblast have a proliferative response to TGFβ1 [20], and that the mouse keratinocyte cell line (BALB/MK) expressing the chimeric estrogen-inducible form of *c-myc*-encoded protein (*myc*ER) suppresses the growth-inhibitory effect of TGFβ1 [19].

As shown in Figure 1, TGFβ1 treatment decreased c-Myc mRNA after 24 h in keratinocytes, while nucleus pulposus cells and articular chondrocytes showed a constant level of c-Myc mRNA. In keratinocytes, we confirmed earlier findings [14,15]. In contrast, nucleus pulposus cells and articular chondrocytes respond differently to TGFβ1 treatment. Although the passage numbers of these cultures are different, we used all of the cultures at the constantly proliferative stage. Considering that keratinocytes has been reported to be growth arrested by TGFβ1 [1], these results suggest that c-Myc mRNA expression correlates with the mitogenic response of the cells to TGFβ1 stimulation. To investigate the effects of c-Myc on cell growth under TGFβ1 stimulation, we inhibited c-Myc function in nucleus pulposus cells using specific inhibitors.

### The mitogenic response to TGFβ1 suppressed by pathway inhibitors

Figure 7a,b indicate that the same levels of endogenous c-Myc protein were detected in nucleus pulposus cells, independent of TGFβ1 treatment. The cell cycle distribution in TGFβ1-treated cells (Figure 5b) indicates a large increase in cells in the S phase, associated with the suppression of p21 and p27 which belong to the Cip/Kip family of cyclin-dependent kinase inhibitors (CKIs) (Figure 7a,b). By contrast, pretreatment with either 10058-F4, a c-Myc, inhibitor or PD98059, an ERK1/2 inhibitor, arrested cell proliferation and cell cycle progression when coexistent with TGFβ1 (Figures 3, 4, 5, 6). Additionally, both inhibitors suppressed c-Myc expression while upregulating p21 and p27 expression (Figure 7c,d) compared to TGFβ1-treated cells (Figure 7b). The elevation of p15, p21 and p27 has been reported to be the main cause of cell cycle arrest by TGFβ1 [29-32]. We therefore analyzed the expression of these three CKIs, but found that p21 and p27 were decreased by TGFβ1, while there was no change in p15 expression (Figure 7). The findings that TGFβ1 did not cause cell cycle arrest in nucleus pulposus cells and that it decreased p21 and p27 expression can be attributed to the sustained c-Myc expression. Previous investigations have suggested the special regulation of CKIs under TGFβ1, mediated by an elevated level of c-Myc [45-47].

### The immediate phosphorylation of ERK1/2 with robust c-Myc expression for 2 h after TGFβ1 treatment

In the time course study, the top panel shows TGFβ1 treatment kept the robust c-Myc expression for 2 h but downregulated it after 6 h (Figure 8a). The downregulation of c-Myc was considered to result from the downregulation of c-Myc mRNA transcription by TGFβ1 through the Smad pathway [16]. As shown in Figure 1b, the level of c-Myc mRNA was downregulated at 60 min and recovered after 240 min. In the protein levels, distinct recovery of c-Myc expression was not detected; nonetheless it was sustained for 24 h. The second panel in Figure 8a shows that TGFβ1 induces the immediate phosphorylation (activation) of ERK1/2; this observation agrees with an earlier study using rat articular chondrocytes by Hirota *et al*. [48]. ERK1 and ERK2 are subtypes of MAPKs activated by a diverse array of extracellular stimuli [49]. The phosphorylation of ERK1/2 in nucleus pulposus cells has been reported to be critical for survival in a hypoxic environment [50]. We also detected marked phosphorylation of ERK1/2 and c-Myc expression in 10% FBS-added cultures. Therefore, growth factors can be considered to drive c-Myc expression and phosphorylation of ERK1/2 in nucleus pulposus cells. However, serum-deprived cells with no supplements (time 0 in Figure 8a) expressed c-Myc, but no phosphorylated ERK1/2. These results suggest that c-Myc itself does not enhance cell growth, but acts as a mediator of exogenous growth stimuli.

### 10058-F4 downregulates c-Myc expression and ERK1/2 phosophorylation

The c-Myc inhibitor 10058-F4 we used was isolated by Yin *et al*. [21] using a yeast two-hybrid system. In order to bind DNA, c-Myc must dimerize with Max. 10058-F4 inhibits c-Myc transcriptional activity by disrupting the c-Myc/Max association. The half-life of Myc is known to be less than 30 min [51]; it has also been reported that the instability of oncoprotein Myc is important to avoid its accumulation in normal cells and that Myc is destroyed by ubiquitin-mediated proteolysis [52]. In this study, we showed almost constant levels of c-Myc mRNA expression in nucleus pulposus cells independent of serum concentrations (Figure 1c) and sustained c-Myc protein expression during treatment with TGFβ1 (Figures 7a,b and 8a). However, inhibition of c-Myc transcriptional activity by 10058-F4 in the presence TGFβ1 resulted in suppression of the mitogenic effect of TGFβ1 (MTT assay (Figure 3) and the cell cycle distribution (Figures 5c, 6)). These results suggest that c-Myc implicates in the effect of TGFβ1. We also observed that 10058-F4 unexpectedly interrupted phosphorylation of ERK1/2 as well as downregulating c-Myc expression (Figure 8c). Because Myc is associated with an extraordinarily large number of genomic sites, it can regulate complex genomic pathways [53-55]. It was also reported that transcriptional response to Myc binding differs markedly according to context and cell type [55]. The elucidation of the role of c-Myc in ERK1/2 phosphorylation in nucleus pulposus cells requires further investigation.

Recent studies investigating 10058-F4 report cell cycle arrest accompanied by suppression of c-Myc mRNA in lymphoma [56] and the suppression of c-Myc with upregulation of levels of p21 and p27 in myeloid leukemia [57,58]. These reports correspond with our observations (Figure 7).

### PD98059 downregulates ERK1 phosphorylation and c-Myc expression

We show that pretreatment with PD98059 significantly blocked the mitogenic and cell cycle promotive effects of TGFβ1 (MTT assay (Figure 4) and cell cycle distribution (Figures 5d, 6)) associated with suppression of c-Myc expression (Figure 7d). In the preliminarily experiments we also examined a protein kinase C inhibitor peptide (19–36) obtained from Calbiochem (Darmstadt, Germany), because inhibition of protein kinase C had been reported to cause abolition of TGFβ1 induced cell growth in rat articular chondrocytes [37], but it did not exert the abolition in nucleus pulposus cells (data not shown). By contrast, PD98059 showed a significant inhibitory effect. PD98059 is an inhibitor for MAP kinase kinases 1 and 2 (MKK), also called MAP/ERK kinases (MEK), the upstream activator of ERK1/2. In the time course study (Figure 8b), the second panel shows only phospho-ERK2 protein bands with the complete absence of phospho-ERK1 for 24 h. The inhibitory effect of PD98059 on MEK2 is known to be less potent than MEK1. The concentration of PD98059 required to give 50% inhibition (IC50) of MEK1 is 4 μM and of MEK2 is 50 μM [22]. Because we used a maximum dose of 30 μM of PD98059, MEK1 was considered to be strongly inhibited. These results suggest that phosphorylated ERK1 is necessary to maintain c-Myc expression and promote cell cycle progression under TGFβ1 stimulation. Our results agree with earlier reports showing that ERK1/2 plays a crucial mediating role in mitogenic signaling of TGFβ1 in mouse BM-MSCs cultured in chondrogenic condition [9] and in rat articular chondrocytes [23].

### Possibility of c-Myc stability supported by phospho-ERK1/2

We showed the persistent expression of c-Myc in nucleus pulposus cells, which are not tumor cells or immortalized cells. As described above, c-Myc appears to be supported by phospho-ERK1/2. Lefevre *et al*. [59] showed that treatment with Raf-1 kinase inhibitor or ERK1/2 inhibitor PD98059 decreased c-Myc production in cultured ocular choroidal melanoma which had a high and constant level of c-Myc. Also, the contribution of Ras/Raf/ERK prevented the rapid degradation of c-Myc by phosphorylation of the serine 62 residue in the N-terminal of c-Myc [24]. They also found that the suppression of glycogen synthase kinase 3 beta (GSK-3β) activity, which phosphorylates threonine 58, a phosphorylation site adjacent to serine 62, enhances c-Myc stability. Although we did not analyze the phosphorylation of c-Myc, these proposed kinetics should be investigated to explain the enhanced stability of c-Myc in nucleus pulposus cells.

Recent investigations have revealed that Myc stability is required in self-renewal and maintenance of murine ES cell pluripotency [44]. These authors evaluated Myc protein levels in ES cells and concluded that elevated Myc activity is able to block the differentiation of multiple cell lineages and that this blocking of differentiation promotes self-renewal. Similarly, c-Myc has been reported to inhibit the terminal stages of adipocyte differentiation [60].

We used cells derived from rat nucleus pulposus of the intervertebral disc to examine how they respond to TGFβ-1 stimulation. Cells constituting the nucleus pulposus are known to be sparse and have a low ability for self-renewal [61]. Although efforts to regenerate disc tissue using cell therapy have accelerated their profiling [62], the precise phenotype of nucleus pulposus cells and their response to various cytokines are still under investigation. In this study, we suggested a specific regulatory pathway of TGFβ1 in which c-Myc and phospho-ERK1/2 play important roles. However, we used the third or fourth passaged culture, which did not contain large notochordal cells. Therefore, some phenotypic change (that is dedifferentiation) may have been induced, as is known to occur for articular chondrocytes. Inevitably, the correlation between differentiation level in the cells and responsiveness to TGFβ1 remains to be elucidated. Moreover, in view of the therapeutic use of TGFβ1 for nucleus pulposus regeneration, the limitation in the use of the rat model needs to be carefully considered. This is because the presence of notochordal cells in the rat coccygeal disc is different from the human situation, in which notochordal cells have been known to disappear after birth. Therefore, future studies using different animal models are necessary to confirm whether the implication of c-Myc and ERK1/2 can generally be attributed to nucleus pulposus cells or it depends on the species of the donor.

## Conclusion

Because our results indicate that both c-Myc and phospho-ERK1/2 are required for proliferation and cell cycle progression, we conclude that the synergistic effect between c-Myc and phospho-ERK1/2 plays a key role in nucleus pulposus cell growth under TGFβ1 stimulation. Therefore, treatment with TGFβ1 should yield different effects depending on the status of these mediators in the target cells.

## Abbreviations

AC: articular chondrocytes; BM-MSCs: bone marrow derived mesenchymal stem cells; BSA: bovine serum albumin; CDK: cyclin dependent kinase; CKIs: cyclin dependent kinase inhibitors; DMEM: Dulbecco's modified Eagle medium; DPBS: Dulbecco's phosphate-buffered saline; ERK1/2: extracellular signal regulated kinase 1/2; FACS: fluorescence-activated cell sorting; FBS: fetal bovine serum; GSK-3β: glycogen synthase kinase-3β; KT: keratinocytes; MAPK: mitogen activated protein kinase; Max: Myc-associated factor X; MEK: MAP/ERK kinase; MEM: minimum essential medium; MKK: MAP kinase kinase; NP: nucleus pulposus; PVDF: polyvinylidene difluoride; RT-PCR: reverse transcriptase-polymerase chain reaction; TBST: Tris-buffered saline/Tween; TGFβ1: transforming growth factor β1; SDS-PAGE: sodium dodecyl sulfate polyacrylamide gel electrophoresis; SEM: standard error of the mean.

## Competing interests

The authors declare that they have no competing interests.

## Authors' contributions

TN and DS conceived of the study and performed the experimental work. DS and JM participate in its design and coordination. TN, DS and JM helped to draft the manuscript. TN and DS performed the statistical analysis. All authors read and approved the final manuscript.
